# Digital Tri-Axis Accelerometer with X/Y-Axial Resonators and Z-Axial Capacitive Seesaw

**DOI:** 10.3390/mi13081174

**Published:** 2022-07-25

**Authors:** Dunzhu Xia, Mohan Yao, Jinhui Li

**Affiliations:** 1 School of Instrument Science and Engineering, Southeast University, Nanjing 210096, China; 220193302@seu.edu.cn (M.Y.); 230198863@seu.edu.cn (J.L.); 2 Key Laboratory of Micro-Inertial Instrument and Advanced Navigation Technology, Ministry of Education, Nanjing 210096, China

**Keywords:** tri-axis accelerometer, resonant accelerometer, seesaw capacitor accelerometer, frequency meter, phase noise

## Abstract

A tri-axis accelerometer with a digital readout circuit and communication system is introduced. It is composed of two resonant accelerometers in the x and y-axis, and a seesaw capacitive one in the z-axis. The device is encapsulated in air to ensure that the z-axis works in an over-damped state. Moreover, the closed-loop drive circuit establishes the x-axis and y-axis in resonant mode, and the z-axis in force balance mode. A miniaturized measurement based on FPGA is designed to collect these output signals. The phase noise of the resonance part and the amplitude noise of the seesaw part are studied by simulation. The model can predict the contribution of each part to the measurement error and Allan variance. Multiplied clock and Kalman filter in sliding window are used to reduce the frequency error. The test results show that the accelerometer has low bias instability (<30 μg), low cross-coupling error (<0.5%), and low nonlinearity (<0.1%). The tri-axis digital accelerometer with serial ports is more valuable than the previous works with large commercial instruments.

## 1. Introduction

A micromachine accelerometer is an important micro inertial navigation sensor. A silicon micro accelerometer has the advantages of small volume, light weight, low cost, high reliability, low power consumption, and mass production. It has important applications in the field of inertial navigation.

The forms of accelerometers include resonant type, capacitive type, piezoresistive type, piezoelectric type, etc. Among them, the capacitance type and resonant type are used more often. According to the structure, the capacitive type can be divided into comb type, seesaw type, and sandwich type. Compared with a capacitive accelerometer, a resonant accelerometer has higher accuracy. There has been a lot of research about resonant accelerometers in recent years [1,2,3,4].

However, a resonant accelerometer needs frequency measurements, and the miniaturization and high speed has always been a difficulty in research. Many resonant accelerometers must be connected to large commercial frequency meters to work. A digital frequency readout system based on FPGA counters is designed to solve the problem, and has a 1 sps sampling rate and 25.9 μHz resolution. The resolution of the whole accelerometer is 71.7 ng, and the bias instability is 1.77 μg [5]. A digital readout circuit based on an AD converter is also designed, whose bias instability can reach 4.3 μg [6]. Due to its complex structure, large size, and high cost, it is difficult to integrate a multi-axis acceleration sensor.

Therefore, to realize the multi-axis integration of acceleration, various forms of three-axis accelerometers were designed. The tri-axis capacitive accelerometer is relatively mature, with middle level precision [7,8]. The research on multi-axis resonant accelerometers is still rare. Its main difficulties focus on the z-axial direction. The performance of the resonant accelerometer is better in the x-axis and y-axis, [9,10] and relatively worse in the z-axis [11]. A capacitive accelerometer is better for the z-axis [12]. A tri-axis accelerometer based on a differential resonant beam and force balance capacitor plate is proposed. It uses a resonant beam to detect the acceleration along the x-axis and y-axis, as well as a force-balanced differential plate capacitor to detect the acceleration along the z-axis [13]. The accelerometer adopts air packaging, which places the z-axis in an over-damping state, and becomes more stable in the force balance mode. The z-axis capacitive accelerometer adopts a sandwich structure. In contrast, the capacitive accelerometer with a seesaw structure is superior in sensitivity and robustness [14]. The seesaw accelerometer is more suitable for the z-axis, for the tri-axis acceleration. Its performance is closer to that of the x-axis and y-axis resonant accelerometer.

A tri-axis silicon micro accelerometer is studied in this paper, with resonators in the x-axis and y-axis, as well as a capacitive seesaw in the z-axis. The micro-electro-mechanical system (MEMS) structure is air-encapsulated, making it easier for the z-axis to achieve force balance. A readout circuit of a multi-axis resonant accelerometer based on FPGA is designed, which realizes the functions of signal readout, frequency measurement, differential compensation coefficient, and data transmission. The tri-axis accelerometer has both high precision and a high readout rate. The system has the advantages of simple structure, small volume, low power consumption, and higher practical value.

## 2. The Structure of Accelerometer System

### 2.1. Structure of the MEMS

**Figure 1:**
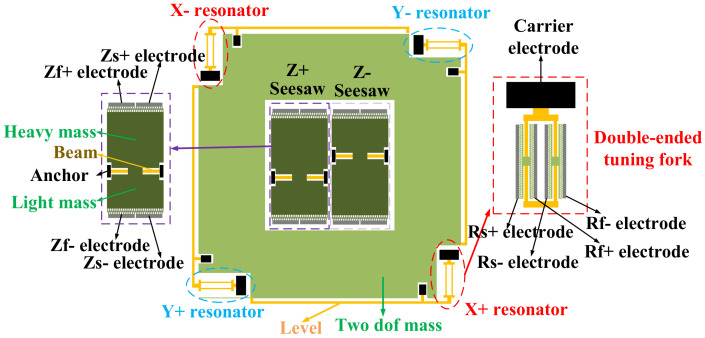
The structure of the accelerometer.

The MEMS structure of the accelerometer is shown in Figure 1. The x-axis and y-axis are resonant in-plane accelerometers, while the z-axis is a seesaw capacitive perpendicular to the MEMS surface. The resonant frequencies in the x-axis and y-axis elements are sensitive to acceleration. The deflection amplitude in the z-axis is sensitive to acceleration. MEMS is encapsulated in air during installation. The x-axis and y-axis share a 2-DOF mass. When there is acceleration on the x-axis, the mass is displaced. The mass pulls the lever, and the lever presses or stretches the resonator, which changes the frequency of the resonator. The changes of the two resonators are opposite, so the output differential signal is proportional to the acceleration. The z-axis uses two differential seesaws. Each seesaw has a light mass and a heavy mass. When there is acceleration on the z-axis, the seesaw rotates, due to the inertia of the heavy mass and the higher moment arm. The acceleration can be obtained by measuring the differential component of the capacitance.

**Figure 2:**
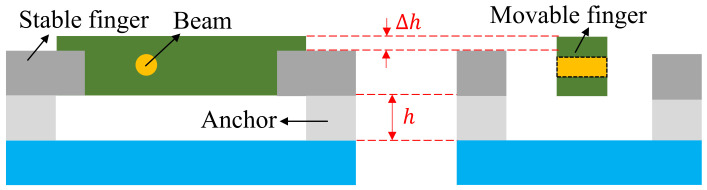
Front view of the comb finger and side view of the comb finger.

Figure 2 is the cross-sectional structure of the comb finger in the z-axis. It features high, movable combs and low, stable combs. The height difference Δ*h* of the comb finger is 10 μm. When the seesaw turns, the differential capacitances decrease on one side and increase on the other side. The thickness of the anchor point is *h* is 30 μm, so it is difficult for the seesaw in the z-axis to touch the substrate, avoiding pull-in.

**Figure 3:**
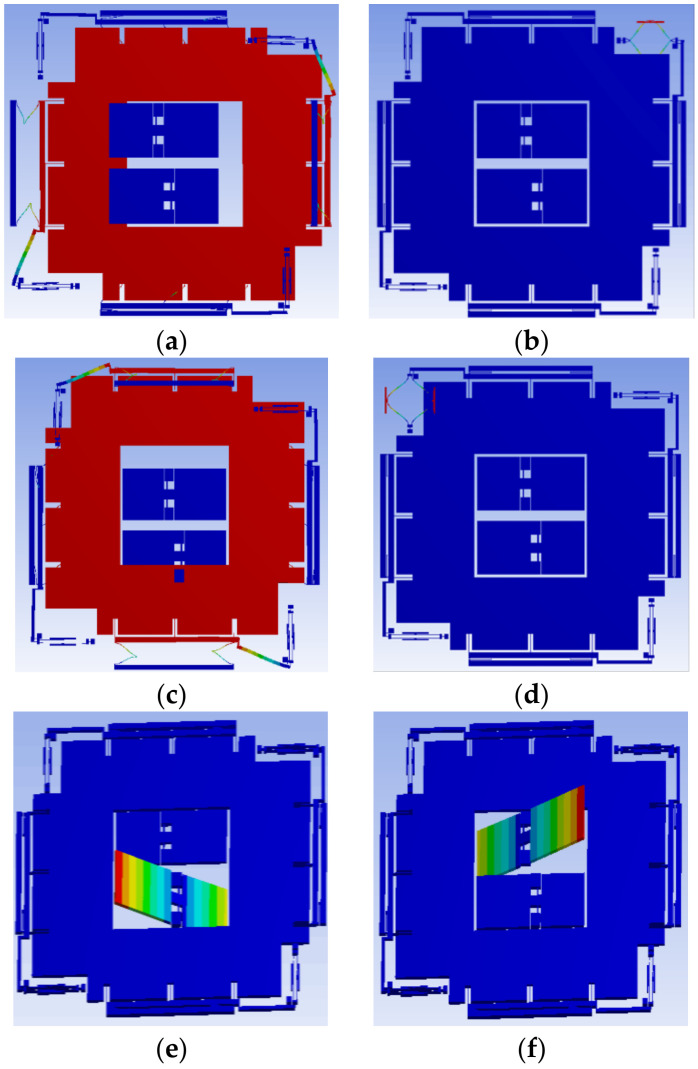
Vibration modal simulation of three-axis acceleration. (**a**) 1 kHz vibration of the mass in the x-axis; (**b**) 20 kHz vibration of a resonator in the x-axis; (**c**) 1 kHz vibration of the mass in the y-axis; (**d**) 20 kHz vibration of a resonator in the y-axis; (**e**) 1 kHz vibration of one seesaw in the z-axis; (**f**) 1 kHz vibration of another seesaw in the z-axis.

Figure 3 is a modal simulation of the accelerometer. The resonant frequencies of the masses are 1 kHz, to ensure that the bandwidth of the accelerometer is several hundred Hz. The vibration of the mass in the x, y, and z axes at 1 kHz is simulated. The x-axis and y-axis resonators are designed at different resonant frequencies around 20 kHz. They are also simulated with vibrations at 20 kHz.

To avoid mutual interference of each resonator, the natural frequencies of each resonator are different frequencies around 20 kHz. Its no-load natural frequency can be expressed as:

(1) $$f_{0}=\frac{1}{2\pi }\cdot \sqrt{\frac{K}{M}}$$

where *K* is the spring constant of the resonant beam, and *M* is the equivalent mass of the resonator. Once an external acceleration is applied to the resonant accelerometer, the natural frequency varies, due to stress change set transferred from lever. The frequency can be rewritten as:

(2) $$f=f_{0}\cdot \sqrt{1+F\frac{0.295L^{2}}{Ehw^{3}}}$$

where *F* is the magnitude of the axial force transferred from the mass, *E* is the elastic modulus, *h* is the thickness of the resonant beam, *L* is the length of the resonant beam, and *w* is the width of the resonant beam. The natural frequencies of the four resonant beams are set as 20,200 Hz, 21,300 Hz, 22,100 Hz, and 23,100 Hz. According to Formula (2), the final lengths of the four resonators can be calculated to be 1220 μm, 1250 μm, 1280 μm, and 1320 μm, respectively. The final structural parameters are shown in Table 1.

### 2.2. Fabrication Process

**Figure 4:**
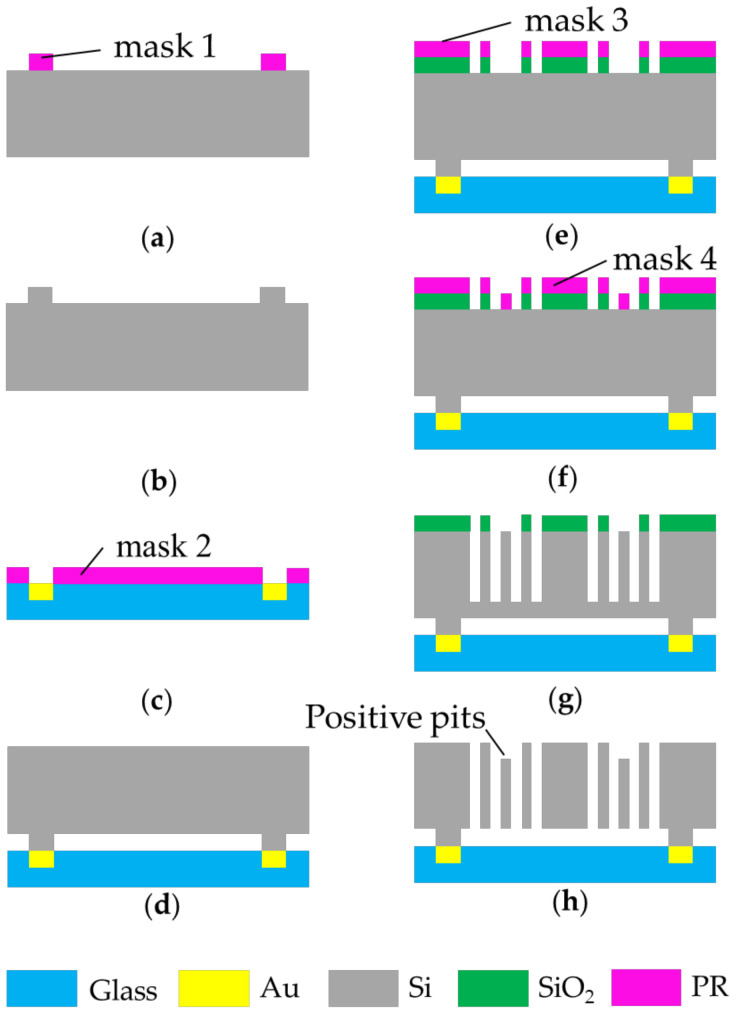
Process flow of three-axis accelerometer. (**a**) Position of anchors determined by mask 1; (**b**) overall etched silicon with ICP; (**c**) Ti and Au deposited on glass; (**d**) anodic bonding; (**e**) position of out-of-plane movable finger determined by mask 3; (**f**) the position of the finger and the through hole defined by mask 4; (**g**) the silicon etched 50μm depth by ICP; (**h**) the positive pits etched by ICP.

Figure 4 shows the fabrication process of this tri-axis accelerometer. The tri-axis accelerometer is fabricated based on a silicon-on-glass (SOG) process. The thickness of the entire silicon wafer is 90 μm. The structure is 60 μm, and its distance from the glass substrate is 30 μm. This pull-in prevention design effectively reduces the processing difficulty, compared with the use of gold plating, to make stops on the substrate. Since the z-axis adopts differential capacitance detection, the comb structure is designed to have unequal heights on one side. Compared with the structure with unequal height on both sides, it avoids the etching of positive and negative pits, and simplifies the fabrication process. It uses four masks to create structures with anchors, decoupling beams, and combs. The specific production process is shown in a previous work [15].

**Figure 5:**
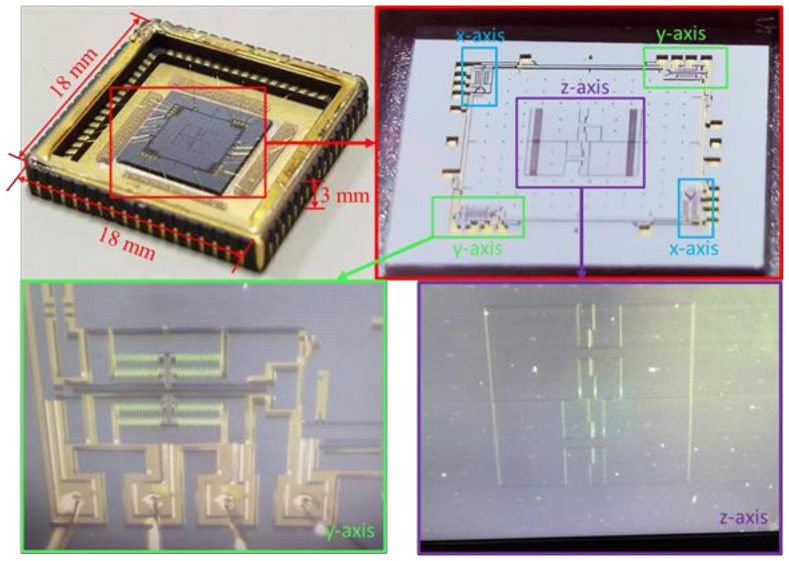
MEMS packaging and internal structure.

The package of the tri-axis accelerometer is shown in Figure 5. The silicon wafer is encapsulated under the air, and the package size is 18 × 18 × 3 mm^3^. In the middle is the seesaw for the z-axis, surrounded by resonators for the x-axis and y-axis.

### 2.3. Structure of the Circuit System

**Figure 6:**
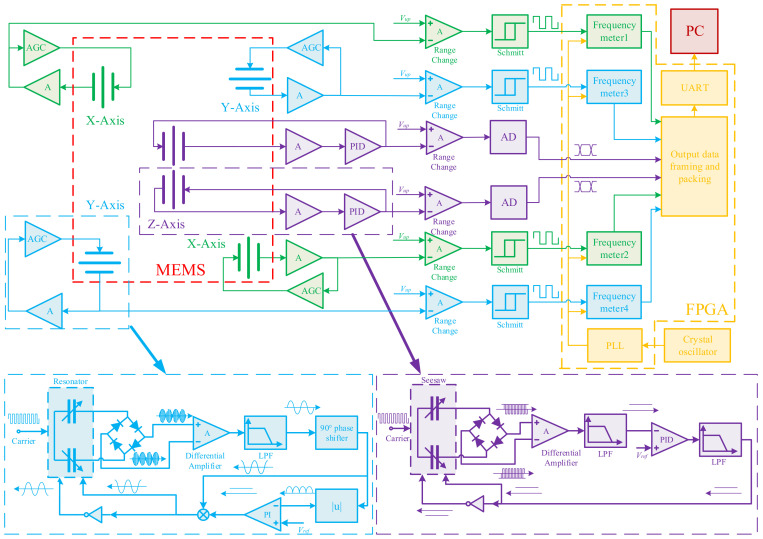
Circuit system of the accelerometers.

The structure of the circuit system is shown in Figure 6. The MEMS part is outlined in red, and the FPGA part is outlined in yellow. The x-axis is shown in green, the y-axis is shown in blue, and the z-axis is shown in purple.

Each accelerometer in the x, y, or z axis has two pairs of differential capacitors in the MEMS structure. Each pair of differential capacitors is equipped with a drive loop. The accelerometers in the x-axis and y-axis work in resonant mode, while the accelerometer in the z-axis works in force balance mode. The x-axis or y-axis loop consists of a resonator, a sensing circuit, an automatic gain control (AGC) circuit, a shaping circuit, and a frequency meter. The differential capacitances change in the resonator when it works. In the sense circuit, the capacitance change is converted into an output voltage signal through the ring diode, differential amplifier, low-pass filter (LPF), and 90° phase shifter. In the AGC circuit, positive feedback is generated by the rectifier and PI controller. The resonator is forced to vibrate at the natural frequency by the feedback force. The output signal is amplified to Schmitt’s working range by the range change amplifier. The output sine wave is converted into a square wave of the same frequency by a Schmitt trigger. The square wave is counted and compared with the reference clock to calculate the frequency.

The z-axis loop contains a seesaw, a sensing circuit, a PID controller, and an AD converter. Driven by the carrier, the change in capacitors is converted into a DC voltage signal by the ring diode. The feedback signal is generated by PID control. The plates are balanced by negative feedback electrostatic forces. The output result is the feedback signal, which is converted into a digital signal by an AD converter, and further processed by the FPGA.

The FPGA part includes four frequency meters, a PLL, a data framing and packing module, and a UART serial port. The PLL multiplies the 50 MHz clock from the crystal oscillator to 200 MHz as the reference for frequency meters. The data widths of the frequencies and voltages are 28 bits and 24 bits, respectively, while the UART sends data of 8 bits. Therefore, data of the frequencies and voltages are framed, packed, and sent to the PC by the UART port.

**Figure 7:**
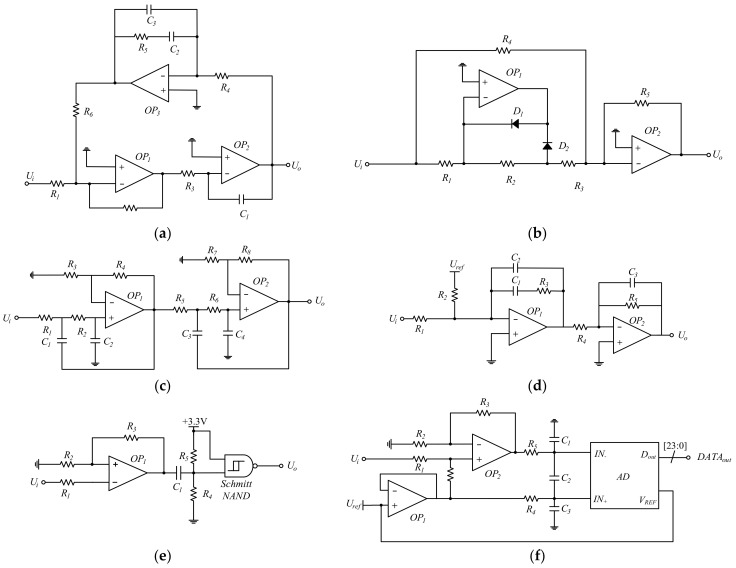
The specific circuit of some modules. (**a**) 90° phase shifter; (**b**) rectifier circuit; (**c**) low-pass filter; (**d**) PI controller; (**e**) range change and shaping circuit; (**f**) range change and AD converter.

Figure 7a is a 90° phase-shifter with three operational amplifiers. It adds feedback control to achieve more precise phase shifting compared to dual operational amplifiers phase shifters. Figure 7b is a rectifier that converts the signal’s negative voltage into positive voltage by two diodes, *D*_1_ and *D*_2_. Figure 7c is a fourth-order Butterworth low-pass filter implemented by two second-order low-pass filters in series. Figure 7d *OP*_1_ is an inverting PI controller, and *OP*_2_ is an inverting amplifier. *R*_3_ is the proportional resistor and *C*_1_ is the integrating capacitor. This circuit compares the input signal *U_i_* with the reference signal *U_ref,_* and outputs a control signal. Figure 7e is a range change and square wave shaping circuit. The signal is amplified by *OP*_1,_ and adjusted to a range suitable for Schmitt trigger operation by pull-up and pull-down resistors. The sine wave can be converted into a square wave of the same frequency by being compared with the +3.3 V voltage in the NAND gate. The duty cycle is adjusted by pull-up and pull-down resistors. Figure 7f is a range change and ADC circuit. *OP*_1_ is a voltage follower, and *OP*_2_ is a single-channel to the differential amplifier. The common mode signal is half of the ADC’s reference voltage. The differential signal is converted into a 24-bit data by the ADC.

### 2.4. Frequency Measurement Based on FPGA

**Figure 8:**
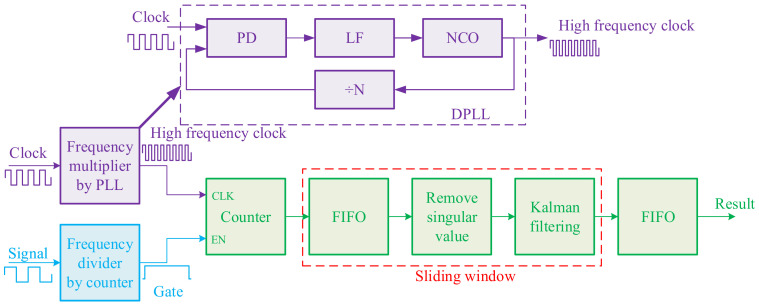
Process of frequency measurement.

The algorithm of the digital frequency meter is shown in Figure 8. The clock from the crystal oscillator is doubled to a high-frequency clock by the digital phase-locked loop (DPLL). The gate is generated in the counter by the frequency divider of the signal.

The gate cycle is $N_{x}$ times the cycle of the signal. The counter counts in the gate to obtain the signal’s frequency. The signal’s frequency is:

(3) $$f_{x}=\frac{N_{x}}{N_{s}}\cdot f_{s}$$

where $N_{s}$ is the count value of the clock, $f_{s}$ is the clock’s frequency, and $f_{x}$ is the signal’s frequency. As the measurement error is a one clock cycle, doubling the clock can greatly reduce the measurement error. After multiplying the clock *n* times, the measurement result is shown in Equation (4).

(4) $$f_{x}=\frac{N_{x}}{nN_{s}}\cdot nf_{s}$$

**Figure 9:**
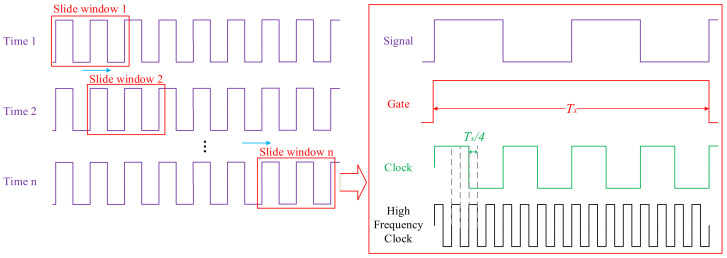
Frequency doubling and sliding window filter method.

The error $\Delta e_{x}$ is shown in Equation (5).

(5) $$\Delta e_{x}=\frac{\Delta f_{x}}{f_{x}}=\frac{1}{nN_{s}}$$

where *n* is the frequency multiplication coefficient. The higher the coefficient, the smaller the measurement error. The sliding window filtering method is shown in Figure 9. The error can be greatly reduced by removing singular values and the Kalman filter.

The value cycle of the sliding window is *n* signal cycles, and the sliding distance is m signal cycles. Since *m* << *n*, the sliding window does not affect the sampling rate. The sampling rate is:

(6) $$SR=\frac{f_{x}}{m}$$

**Figure 10:**
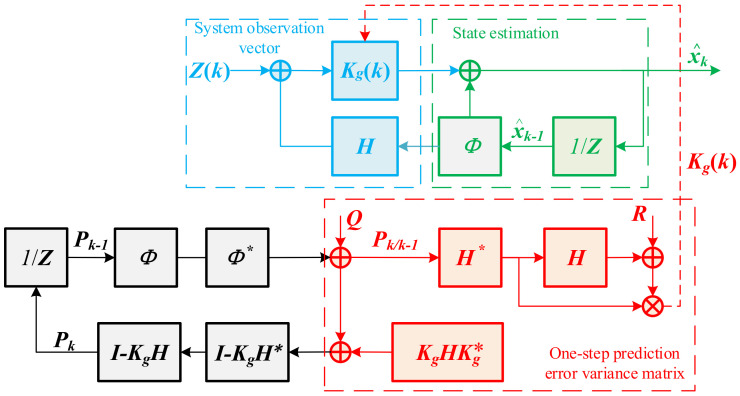
The flow diagram of Kalman filter algorithm.

Parameters can be changed as needed. While they are set to *m* = 220, *n* = 22,000, the sampling rate is 100 sps, with a hysteresis of 1 s. The flow diagram of the Kalman filter algorithm is shown in Figure 10. It was found to filter out the noise in the accelerometer and improve the accuracy in previous work [16].

**Figure 11:**
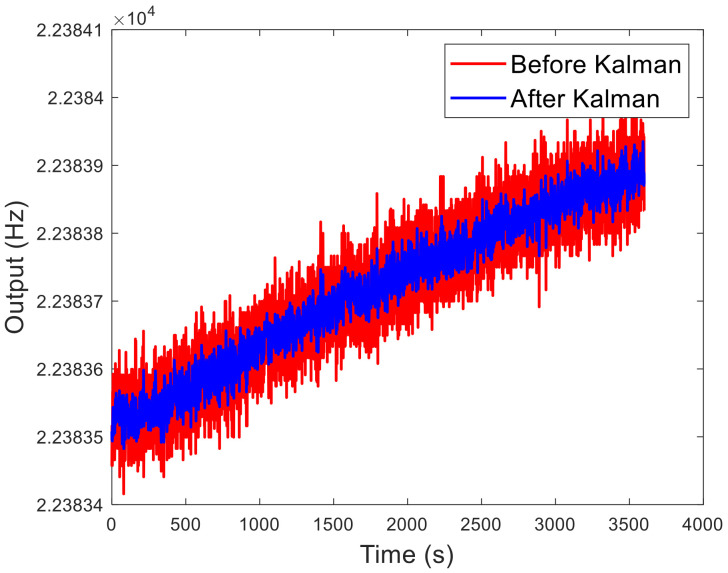
Influence of Kalman filter on signal quality.

***X_k_*** is the state vector, which is the output. ***Z_k_*** is the observation vector, which is the input. ***Φ_k,k_*_−1_** is the one-step state transition matrix. ***K_g_*** is the Kalman gain. ***H_k_*** is the observation matrix. ***R_k_*** is the observation noise. ***Q_k_*** is the process noise. Parameters are chosen as ***R*** = 0.1 and ***Q*** = 10^−5^. The result is shown in Figure 11. Noise is improved in the short term, and the signal is not significantly delayed. So, the sliding window filtering method can significantly improve the measurement accuracy.

## 3. Noise and Error Analysis

### 3.1. Phase Noise of X-Axis and Y-Axis

**Figure 12:**
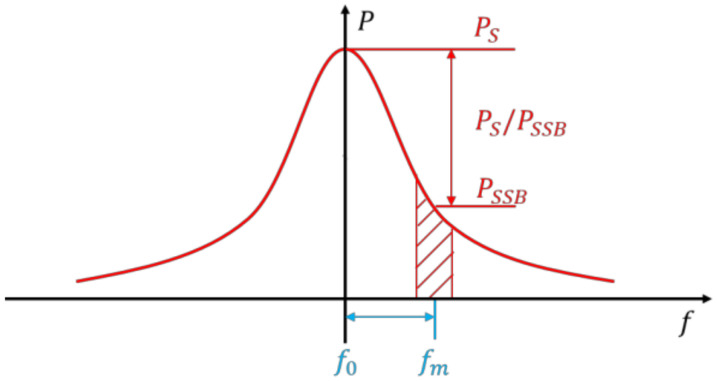
Definition of phase noise.

The phase noise of a resonant accelerometer causes frequency instability and measurement error. Therefore, it is necessary to calculate the phase noise of each module, and put forward the optimization scheme. The model and simulation method of phase noise were studied by predecessors [17]. As shown in Figure 12, according to the definition of American National Bureau of standards, phase noise can be defined by Equation (7).

(7) $$S_{\varphi }\left(f_{m}\right)=\frac{P_{SSB}\left(f_{0}+f_{m},1\mathrm{Hz}\right)}{P_{S}}$$

The phase noise of the single sideband $S_{\varphi }\left(f_{m}\right)$ is the ratio of the power $P_{SSB}$ of one phase modulation sideband to the total carrier power $P_{S}$ within 1 Hz bandwidth at the deviation from the carrier power $f_{m}$, and the unit is dBc/Hz. The irrelevant noise near the carrier frequency, amplitude noise, and phase noise account for half, respectively. Bringing this into the above equation achieves:

(8) $$S_{\varphi }\left(f_{m}\right)=\frac{\bar{v_{n}^{2}}\left(f_{0}+f_{m},1\mathrm{Hz}\right)/2}{v_{pk}^{2}/2}$$

The drive circuit is composed of operational amplifiers (op2177), resistors, and capacitors. Since the voltage noise of the amplifier op2177 is constant over the entire spectrum, the phase noise can be expressed in Equation (9). The white noise value of op2177 is 0.2 μV(RMS).

(9) $$n_{r}=S_{\varphi }\left(f_{m}\right)=\frac{\bar{v_{n}^{2}}}{v_{pk}^{2}}$$

**Figure 13:**
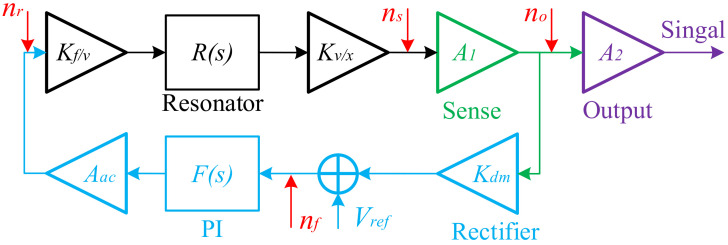
Noise source of x-axis and y-axis.

The demodulated noise transfer loop is shown in Figure 13 [18]. The transfer function of the resonator is:

(10) $$R\left(s\right)=\frac{1}{2m\omega_{n}}\cdot \frac{1}{s+\frac{\omega_{n}}{2Q}}$$

The transfer function of the sensitive circuit is *A*_1_. The transfer function of the feedback circuit is:

(11) $$F\left(s\right)=K+\frac{K_{i}}{s}$$

The transfer function of the output amplifier is *A*_2_. The equivalent gain of the AC voltage is *A_ac_*, and the equivalent gain of the demodulator is *K_dm_*. The demodulation function is performed by the rectifier (absolute value generator and the LPF in PI controller).

The transfer function of mechanical noise of the resonator is:

(12) $$NTF_{r}=\frac{A_{1}A_{2}K_{f/v}K_{v/x}R(s)}{1-A_{1}A_{ac}K_{dm}K_{f/v}K_{v/x}R(s)F(s)}$$

The transfer function of electrical noise of the detection circuit is:

(13) $$NTF_{s}=\frac{A_{1}A_{2}}{1-A_{1}A_{ac}K_{dm}K_{f/v}K_{v/x}R(s)F(s)}$$

The transfer function of electrical noise of the feedback circuit is:

(14) $$NTF_{f}=\frac{A_{1}A_{2}R(s)F(s)}{1-A_{1}R(s)F(s)}\cdot \frac{A_{1}A_{2}K_{dm}K_{f/v}K_{v/x}R(s)F(s)}{1-A_{1}A_{ac}K_{dm}K_{f/v}K_{v/x}R(s)F(s)}$$

The transfer function of electrical noise of the output amplifier circuit is:

(15) $$NTF_{o}=A_{2}$$

The total noise of closed-loop system is the sum of the four, and the results are concluded in Equation (16). The absolute value simplified expressions of these transfer functions are shown in Table 2.

(16) $$S_{\omega }=NTF_{r}^{2}\frac{\bar{v_{nr}^{2}}}{v_{pk}^{2}}+NTF_{s}^{2}\frac{\bar{v_{ns}^{2}}}{v_{pk}^{2}}+NTF_{f}^{2}\frac{\bar{v_{nf}^{2}}}{v_{pk}^{2}}+A_{2}^{2}\frac{\bar{v_{no}^{2}}}{v_{pk}^{2}}$$

**Figure 14:**
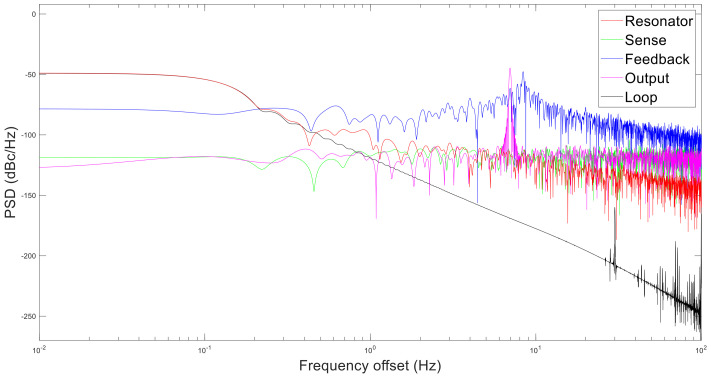
Phase noise of each part in simulation.

Then, the simulation parameters are shown in Table 3. The simulation result is shown in Figure 14.

The phase noise decreases with the increase in frequency offset. The phase noise contribution from high to low is feedback circuit, sense circuit, output circuit, and resonator. In addition, the loop itself is unstable, which also contributes some noise. This is because the noise of feedback circuit is amplified through most parts. Therefore, the feedback circuit should be optimized to reduce the total noise. L(f) is the phase noise spectrum, which is the form of $S_{\omega }$ in dB.

(17) $$L(f)=10\log[S_{\omega }(f)]$$

The periodic jitter caused by the phase noise of the loop is $J_{PER\_loop}$. The relationship between the periodic jitter $J_{PER\_loop}$ and the phase noise spectrum L(f) is:

(18) $$RMS\;J_{PER\_loop}=\frac{\sqrt{\theta^{2}\left(t\right)}}{2\pi f_{c}}=\frac{1}{2\pi f_{c}}\sqrt{2\int_{0}^{\infty }10^{\frac{L\left(f\right)}{10}}df}$$

The total phase jitter of the whole drive circuit is 73.92 ns.

### 3.2. Other Frequency Errors of X-Axis and Y-Axis

In addition to the phase noise of the loop, the Schmitt trigger phase jitter *J_PER_* and crystal frequency errors Δ*e_x_* also contribute to the frequency measurement error. In the time domain, any frequency signal can be expressed in Equation (19).

(19) $$V\left(t\right)=\left[A+\varepsilon \left(t\right)\right]\sin\left[2\pi ft+\varphi +\Delta \varphi \left(t\right)\right]$$

**Figure 15:**
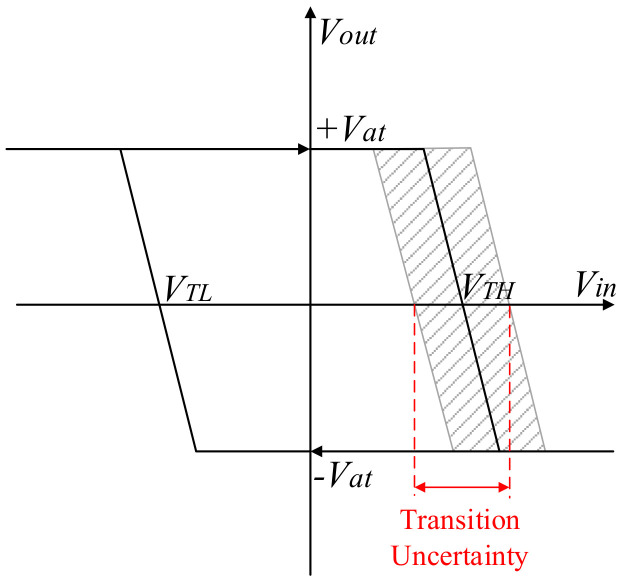
First-order model with input offset voltage and noise.

The jump delay of the Schmitt trigger is shown in Figure 15.

The uncertain delay of the Schmitt trigger produces phase jitter. $T_{PER}$ is set as the actual delay time, and $T_{0}$ is set as the ideal delay time. The time difference $J_{PER}$ between them is expressed in Equation (20).

(20) $$J_{PER}=T_{PER}-T_{0}$$

The magnitude of phase jitter can be described by standard deviation in Equation (21).

(21) $$RMS\;J_{PER}=\sqrt{J_{PER}^{2}}$$

The gate generated from the signal divided by the counter eliminates the ±l cycle error of the signal. However, it produces the ±l cycle error of the clock. After *n* times frequency multiplication, the count result is:

(22) $$f_{x}=\frac{N_{x}}{nN_{s}\pm 1}\cdot nf_{s}$$

The counting error is:

(23) $$\Delta f_{x}=\frac{N_{x}}{nN_{s}}\cdot \frac{1}{nN_{s}\pm 1}nf_{s}+\frac{1}{nN_{s}\pm 1}\cdot n\Delta f_{s}$$

As $nN_{s}\gg 1$,

(24) $$\Delta f_{x}\approx \frac{N_{x}}{nN_{s}^{2}}\cdot f_{s}+\frac{1}{N_{s}}\cdot \Delta f_{s}$$

The absolute error of signal from the crystal is $\Delta f_{s}$ and the relative error $\Delta e_{x}$ is shown in Equation (25).

(25) $$\Delta e_{x}=\frac{\Delta f_{x}}{f_{x}}=\frac{N_{x}}{nN_{s}^{2}}\cdot \frac{f_{s}}{f_{x}}+\frac{1}{N_{s}}\cdot \frac{\Delta f_{s}}{f_{x}}=\frac{1}{nN_{s}}+\frac{\Delta e_{s}}{N_{x}}$$

The error in one signal period is discussed. The measurement errors from the Schmitt triggers and crystal oscillators are calculated by the data sheets. The delay of the Schmitt trigger is 190 ns, so the phase jitter is 51.075 ns. The frequency instability of the crystal oscillator is 50 ppm, so $\Delta e_{x}$ is 50 × 10^−6^.

### 3.3. Prediction of Allan Deviations of X-Axis and Y-Axis

The final relative error of the frequency measurement can be expressed as:

(26) $$e=F_{x}\cdot J_{PER\_loop}+F_{x}\cdot J_{PER}+\Delta e_{x}$$

where $F_{x}\cdot J_{PER\_loop}$ is the error caused by the drive circuit, $F_{x}\cdot J_{PER}$ is caused by Schmitt, and $\Delta e_{x}$ is caused by the crystal. The contribution of each part to the error is shown in Table 4.

The relationship between frequency noise and phase noise is:

(27) $$S_{y}(f)=\frac{f^{2}}{v_{0}^{2}}S_{\varphi }(f)$$

The Allan variance formula is equivalent to a transfer function; the Allan variance can be expressed as:

(28) $$\sigma_{y}^{2}(\tau )=\int_{0}^{\infty }S_{y}^{}(f){\left|H_{A}(jf)\right|}^{2}df$$

where the transfer function is:

(29) $${\left|H_{A}(jf)\right|}^{2}=2\frac{\sin^{2}(\pi \tau f)}{{\left(\pi \tau f\right)}^{2}}$$

**Figure 16:**
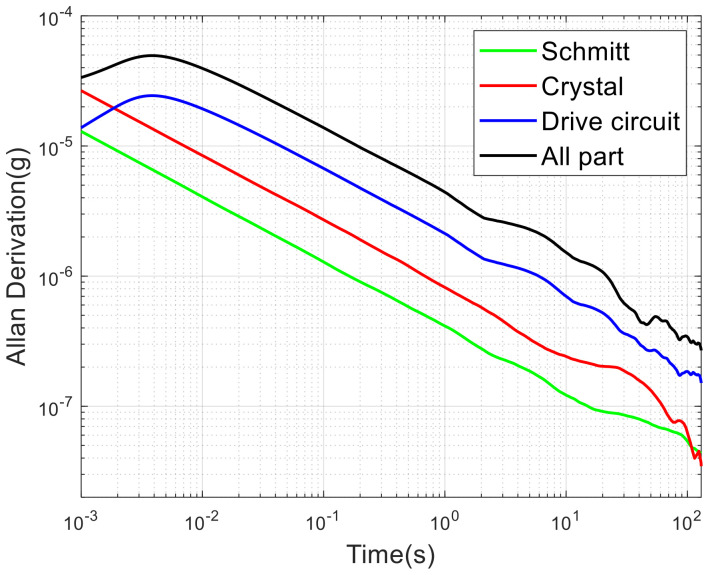
Allan deviation in x-axis and y-axis by simulation.

The Allan deviations produced by each section are shown in Figure 16. This is slightly different from the error result. Since the simulation cannot add temperature drift characteristics, the Allan curve has no upturned segment. The hierarchy of error contribution from large to small is drive circuit, Schmitt trigger, and crystal. Therefore, according to the equal action principle, it should select a lower phase noise drive circuit and a higher speed Schmitt trigger. It is unnecessary to replace the crystal oscillator. 

### 3.4. Amplitude Noise of the Z-Axis

**Figure 17:**
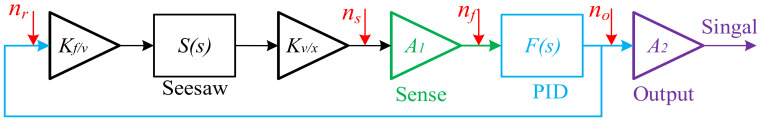
Simulation of phase noise in z-axis system.

The z-axis simulation system is shown in Figure 17. The transfer function of the seesaw is *S*(*s*). The transfer function of the PID is *F*(*s*).

They are represented by the Equations (30) and (31). The z-axis is almost stationary at very low frequencies.

(30) $$S\left(s\right)=\frac{m^{-1}}{s^{2}+\frac{\omega_{n}s}{Q}+\omega_{n}^{2}}$$

(31) $$F\left(s\right)=K+\frac{K_{i}}{s}+K_{d}s$$

The $n_{r}$ is the mechanical noise of the resonator, $n_{s}$ is the electrical noise of the differential amplification circuit, $n_{f}$ is the electrical noise of the feedback circuit, and $n_{o}$ is the electrical noise of the output amplification circuit. The z-axis simulation parameters are shown in Table 5.

**Figure 18:**
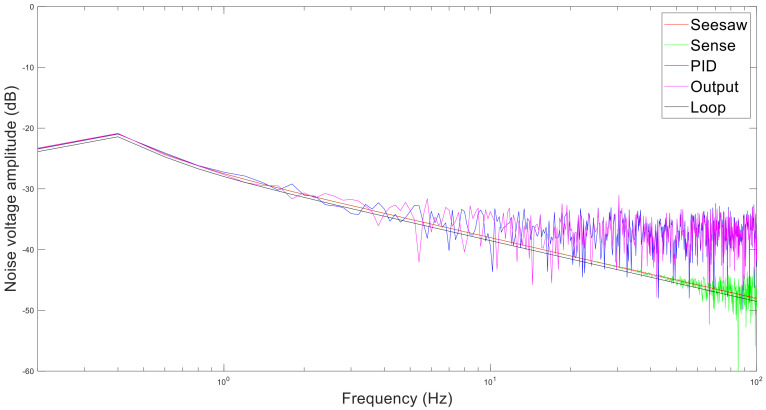
Noise spectrum of z-axis.

The noise spectrum is chosen at 0–100 Hz because the z-axis works in force balance mode, and outputs direct voltage. By Fourier transform, the noise spectrum of the z-axis is shown in Figure 18. The noise power also decreases with the increase in frequency. The voltage stability within five seconds is shown in Table 6. The seesaw, PID, and output circuit generate larger noise than the resonator and sense circuit.

## 4. Experimental Performance of the System

### 4.1. Test Environment of Equipment

**Figure 19:**
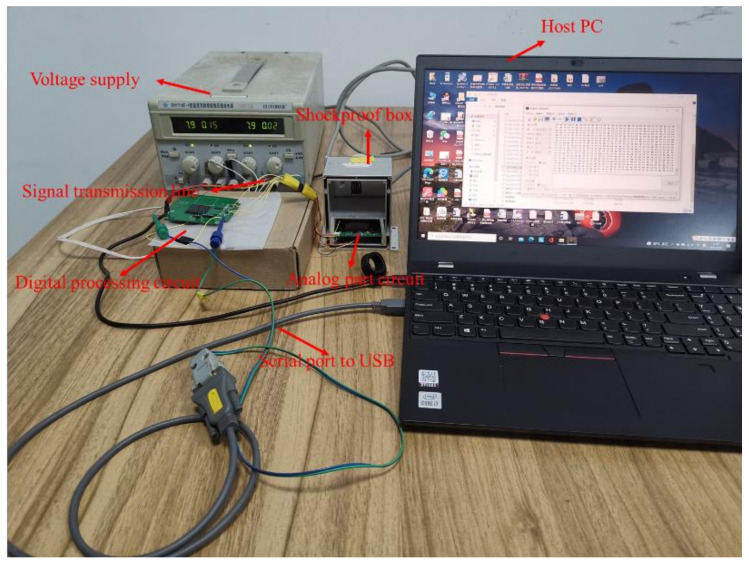
Photo of test environment of tri-axis accelerometer.

The environment of the static test is shown in Figure 19. The device consists of an analog board (55 mm × 55 mm), a digital board (65 mm × 65 mm), a shockproof box, and a host computer. The analog board is packed in a shockproof box to avoid being affected by external vibration. The output signal of the analog board is transmitted to the digital board through the signal transmission line. UART transmits the data to the upper computer through the transfer line.

The system is supplied with a voltage of ±8 V by a constant voltage power supply. The positive current is 0.15 A, and the negative voltage current is 0.01 A. The total power consumption is 1.36 W. The power consumed by the analog part is 0.32 W, while the digital part is 1.04 W. This is due to the high operating frequency of the FPGA (200 MHz) and the aging process (65 nm). The application of the new FPGA can greatly reduce power consumption.

### 4.2. Sensitivity and Compensation Factor

The six sides of the box are then turned up so that the x, y, and z axes are under acceleration of ±1 g. The data are read, and the scale factor calculated. Since the output drifts over time, the difference between the two resonators needs to be calculated to cancel it out. For a single-axis resonant accelerometer, the scale factors of the two differential resonators are not exactly the same. Therefore, it is necessary to find a compensation coefficient k to minimize the variance of the frequency difference $F_{dif}$, as shown in Equation (32). The compensated scale factor *SF* is represented by Equation (33), where *SF*_+_ and *SF*_−_ are the original scale factors of two differential resonators.

(32) $$\left\{\begin{array}{c} F_{dif}\left(k\right)=\frac{F_{+}-kF_{-}}{1+k}\\ F_{dif\_sf}=min\left(\bar{F_{dif}{\left(k\right)}^{2}}\right) \end{array}\right.$$

(33) $$SF\left(k\right)=\frac{SF_{+}+kSF_{-}}{1+k}$$

The compensation coefficients for the x, y, and z axes are 1.25, 1.01, and 1.3000, respectively. The compensated scales are 108.72 Hz/g, 106.89 Hz/g, and 0.9836 V/g, respectively.

### 4.3. Linearity and Range

**Figure 20:**
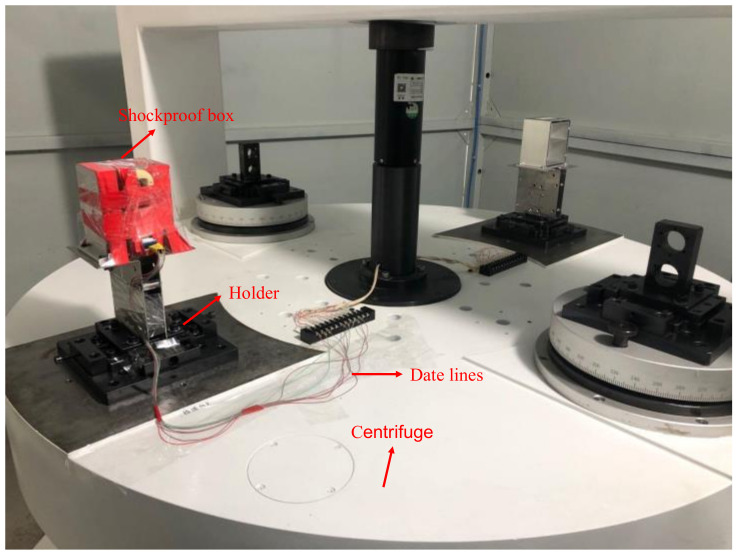
Dynamic test setup on the centrifuge.

The device is mounted on a centrifuge to measure range and linearity. The environment of the dynamic test is shown in Figure 20. The acceleration applied by the centrifuge to the device is 0, ±0.1 g, ±0.2 g, ±0.5 g, ±1 g, ±2 g, ±5 g, and ±10 g. The frequency difference of the output is measured, and a straight line is fitted.

**Figure 21:**
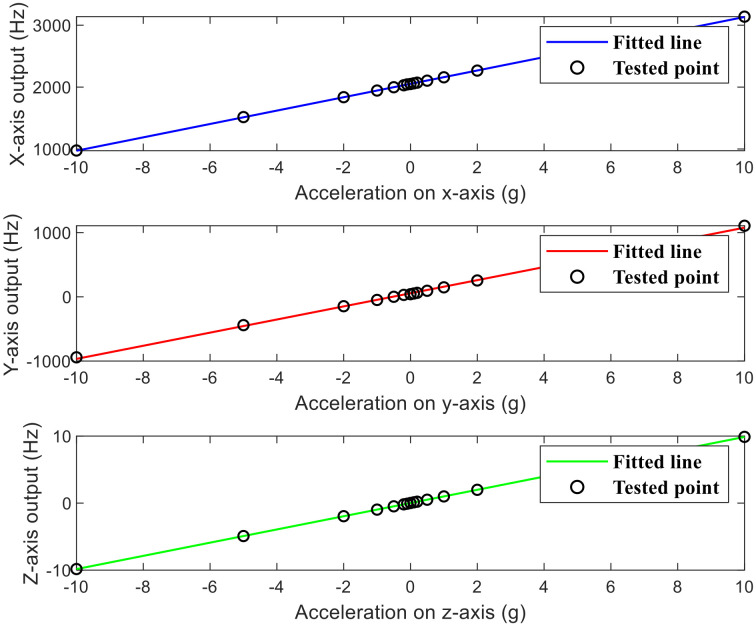
Linearity and range of tri-axis accelerometer.

The result is shown in Figure 21. The nonlinearities of the x-axis, y-axis, and z-axis are 0.0821%, 0.0989%, and 0.0626%, respectively.

### 4.4. Bias Instability

**Figure 22:**
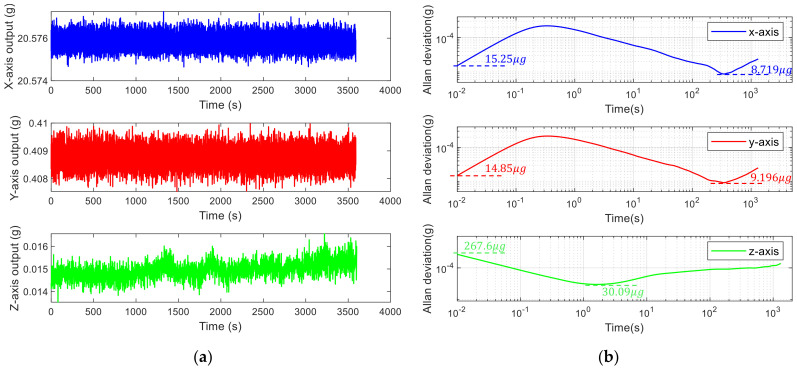
The output of the tri-axis accelerometer within 1 h at 100 sps. (**a**) Time domain plot of zero output; (**b**) Allan variance.

When the sampling rate of the x-axis, y-axis and z-axis is 100 sps, the outputs within 1 h are shown in Figure 22a. Due to processing errors, the two resonators have different coefficients of drift over time. So, the zero bias cannot be completely zeroed. However, the zero bias can be calculated after a test, and zeroed in the FPGA. Output without zero offset can be obtained in the next test. The Allan variance is shown in Figure 22b. The specific data are shown in Table 7. At 100 sps, the Allan deviation of the x-axis, y-axis, and z-axis is 15.25 μg, 14.85 μg, and 267.6 μg, respectively. The minimum Allan variances of the x-axis is 8.7193 μg at 345 s, the y-axis is 9.5013 μg at 350 s, and the z-axis is 30.093 μg at 1.61 s. The Allan variances of the x-axis and y-axis have an upturn before 0.2 s. This is due to the Kalman filter reducing noise in the short term. But it does not improve the long-term stability of the signal. Therefore, the initial segment of the Allan curve is depressed, and the phenomenon of upturning appears. Quantization noise can also be obtained from the curve of the Allan deviation. The quantization noises of the x, y, and z axes are 3.5262 μg, 3.5013 μg, and 1.6941 μg, respectively.

### 4.5. Cross-Axis Coupling Errors

The calculation method of the cross-coupling coefficient is shown in Formula (34). $K_{xy}$ is the coupling coefficient of the y-axis on the x-axis. $K_{xz}$ is that of the z-axis on the x-axis. $K_{yx}$ is that of the x-axis on the y-axis. $K_{yz}$ is that of the z-axis on the y-axis. $K_{zx}$ is that of the x-axis on the z-axis. $K_{zy}$ is that of the y-axis on the z-axis. Placing the six sides of the device horizontally, the output of each channel can be obtained when the x, y, and z axes are ±1 g. Cross-coupling coefficients between the various axes can be calculated.

(34) $$\left[\begin{array}{ccc} K_{xx} & K_{yx} & K_{zx}\\ K_{xy} & K_{yy} & K_{zy}\\ K_{xz} & K_{yz} & K_{zz} \end{array}\right]=\left[\begin{array}{ccc} 1 & \frac{X_{+1g}-X_{-1g}}{2} & \frac{X_{+1g}-X_{-1g}}{2}\\ \frac{Y_{+1g}-Y_{-1g}}{2} & 1 & \frac{Y_{+1g}-Y_{-1g}}{2}\\ \frac{Z_{+1g}-Z_{-1g}}{2} & \frac{Z_{+1g}-Z_{-1g}}{2} & 1 \end{array}\right]$$

**Figure 23:**
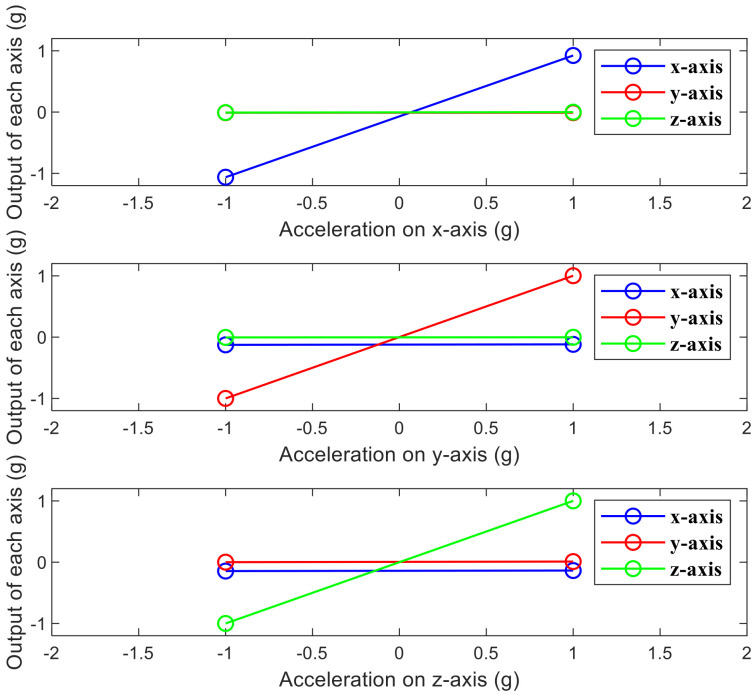
Cross-axis coupling errors test of the tri-axis accelerometer.

The test results of the cross-axis coupling errors are shown in Figure 23. The cross-coupling coefficients between the axis and the test results are shown in Table 7.

### 4.6. Comparison with Other Works

Table 8 lists the performances of several accelerometers, and compares them with the experimental results in this paper. The performance of the partially vacuum-encapsulated uniaxial resonant accelerometer is better than that of the accelerometer in this paper. Simple construction and high Q-factor makes them less noisy. However, when it comes to multi-axis resonant accelerometers, their performance is much worse. Our work has advantages over in-plane x-axis and y-axis resonant accelerometers in noise and bias stability. The seesaw capacitive accelerometer has advantages over the resonant one in the z-axis. Moreover, the zero bias stability and noise of our work are obviously better than the digital tri-axis capacitive accelerometer.

## 5. Conclusions

This paper presents a digital tri-axis accelerometer. The x-axis and y-axis are resonant, and the z-axis is the seesaw capacitance. A miniaturized digital measurement system based on FPGA is designed, which realizes four-channel frequency measurement and two-channel voltage measurement. Compared with commercial frequency meter and multimeter, the system has small volume and power consumption, many measurement channels, and fast measurement speed. Obviously, the miniaturized digital measurement system designed in this paper is more suitable for a multi-axis accelerometer. The source of the phase error of the resonant accelerometer and its influence on frequency stability are analyzed. Some improved methods are discussed and verified. It is effective to reduce frequency jitter, which lowers the noise drive circuit, high-speed Schmitt trigger, and Kalman filter in sliding window. In the case of large noise in the analog part, the effect of using a thermostatic crystal oscillator is not good. In this paper, the amplitude error and instability of a seesaw capacitive accelerometer are analyzed. The driving of the accelerometer is realized on an analog board, and the measurement is realized on a digital board. The tri-axis accelerometer is evaluated by test. The scale factors of the x-axis, y-axis, and z-axis are 108.72 Hz/g, 106.89 Hz/g, and 0.9836 V/g, respectively. The quantization noise is 3.5262 μg, 3.5013 μg, and 1.6941 μg, respectively. The bias instability is 8.7193 μg, 9.1956 μg, and 30.093 μg, respectively. Nonlinearity is 0.0821%, 0.0989%, and 0.0626%, respectively. The cross-coupling error is less than 0.5%. Comparing the resonant accelerometer with the seesaw capacitive accelerometer, it is found that the performance of the resonant accelerometer is more stable. This is due to the small phase and large amplitude component of noise. However, due to the problems of MEMS processing technology, the performance of the resonant z-axis accelerometer is not as good as the seesaw capacitive accelerometer. Therefore, the x-axis and y-axis are resonance, and the z-axis is seesaw, in order to improve the performance of the z-axis accelerometer. In future work, we will optimize and simplify the structure of accelerometers and readout circuits, in order to improve the accuracy and sampling rate.

## Figures and Tables

**Table 1:** Structure parameter of the tri-axis accelerometer.

Parameters	Size	Unit
Overall thickness	90	μm
Structure thickness	60	μm
Anchor thickness	30	μm
Area of the 2-DOF mass	69.8	mm^2^
Length of the decoupling beam	1300	μm
Width of the decoupling beam	20	μm
Length of the restraining beam	800	μm
Width of the restraining beam	15	μm
Lever ratio	3070:10	μm:μm
Width of the resonant beam	8	μm
Length of the resonant beam	1320/1280/1250/1220	μm
Length of the seesaw beam	300	μm
Width of the seesaw beam	50	μm
Area of the heavy mass	2000 × 2800	μm × μm
Area of the light mass	2000 × 1600	μm × μm

**Table 2:** The performance of partial vacuum description of system noise.

Noise	Value	System Transfer Function	Source
$n_{r}$	$\frac{\bar{v_{nr}^{2}}}{v_{pk}^{2}}$	$|NTF_{r}|\approx \frac{A_{2}}{A_{ac}K_{dm}F}$	Mechanical noise of resonator
$n_{s}$	$\frac{\bar{v_{ns}^{2}}}{v_{pk}^{2}}$	$|NTF_{s}|=\frac{A_{2}}{A_{ac}K_{dm}K_{f/v}K_{v/x}RF}$	Electrical noise of sense circuit
$n_{f}$	$\frac{\bar{v_{nf}^{2}}}{v_{pk}^{2}}$	$|NTF_{f}|=A_{2}$	Electrical noise of AGC
$n_{o}$	$\frac{\bar{v_{no}^{2}}}{v_{pk}^{2}}$	$|NTF_{o}|=A_{2}$	Electrical noise of output amplifier circuit

**Table 3:** Parameters of simulation of x-axis and y-axis.

Parameter	Value	Unit	Parameter	Value	Unit
$K_{v/x}$	0.25	V/m	Q	80	-
$K_{f/v}$	5 × 10^−8^	N/V	*m*	10^−8^ kg	kg
$A_{1}$	5 × 10^5^	-	K	10,000	-
$A_{2}$	3	-	$K_{i}$	10,000	-
$\omega_{n}$	138,160	rad/s	$n_{r}$,$n_{s}$,$n_{f}$,$n_{o}$	2 × 10^−7^	V

**Table 4:** Relative errors of frequency caused by surface crystal and Schmitt trigger.

Parameter	Value
$F_{x}\cdot J_{PER\_loop}$	1.4784 × 10^−4^,
$F_{x}\cdot J_{PER}$	1.0280 × 10^−4^
$\Delta e_{x}$	0.5 × 10^−4^
e	3.0064 × 10^−4^

**Table 5:** Parameters of simulation of z-axis.

Parameter	Value	Unit	Parameter	Value	Unit
$K_{v/x}$	0.01	V/m	***Q***	1	-
$K_{f/v}$	5 × 10^−8^	N/V	***m***	10^−9^	kg
$A_{1}$	200	-	***K***	10	-
$A_{2}$	1.25	-	$K_{i}$	100	-
$\omega_{n}$	6280	rad/s	$K_{d}$	10	-

**Table 6:** Simulation results of z-axis.

Parameter	Value	Unit
Duration	5	s
Step	10^−6^	s
Average voltage	2.50044	V
Voltage stability	0.1967	mV

**Table 7:** The experiment results of the tri-axis accelerometer.

Parameter	x-Axis	y-Axis	z-Axis
Compensation coefficient	1.25	1.01	1.3000
Scale factor	108.72 Hz/g	106.89 Hz/g	0.9836 V/g
Q-factor	80	80	<1
Resonant frequency	21.598 kHz/2.2382 kHz	21.975 kHz/22.116 kHz	-
Sampling rate	100 sps	100 sps	100 sps
Output jitter	0.94796 mHz	0.98016 mHz	29.599 μV
Zero bias	20.5759 g	0.4088 g	0.0150 g
Zero bias instability	8.7193 μg	9.1956 μg	30.093 μg
Quantization noise	3.5262 μg	3.5013 μg	1.6941 μg
Range	±10 g	±10 g	±10 g
Nonlinearity	0.0821%	0.0989%	0.0626%
Cross-coupling coefficients	$K_{xy}$ = 0.0017% $K_{xz}$ = 0.35%	$K_{yx}$ = 0.39% $K_{yz}$ = 0.10%	$K_{zx}$ = 0.43% $K_{zy}$ = 0.48%

**Table 8:** Comparison with other resonant accelerometers.

Parameters	[5]	[6]	[19]	[20]	[21]	[13]	This Work
Type	Resonant	Resonant	Capacitive	Capacitive	Resonant	Resonant /plate	Resonant /seesaw
Axis	Single	Single	Three	Three	Three	Three	Three
Signal type	Digital	Digital	Digital	Digital	Analog	Analog	Digital
Scale factor	361 Hz/g	5.09	X: 34.12 mV/g Y: 43.78 mV/g Z: 96.37 mV/g	X: 170 mV/g Y: 170 mV/g Z: 26 mV/g	X: 52.57 Hz/g, Y: 51.64 Hz/g, Z: 31.65 Hz/g	X: 21.30 Hz/g, Y: 12.24 Hz/g, Z: −3.341 V/g	X: 108.72 Hz/g, Y: 106.89 Hz/g, Z: 0.9836 V/g
Frequency	20 kHz	3625	18.5 kHz, 18.5 kHz, 24.8 kHz	-	25 kHz, 28 kHz, 10 kHz	70 kHz/-	22 kHz/-
Q-factor	300,000	4231	-	-	-	-	X/Y: 80 Z: <1
Bias stability	0.055 μg	4.3 μg	X: 560.90 μg Y: 428.16 μg Z: 145.15 μg	X: 3.8 mg Y: 3.8 mg Z: 5.4 mg	X: 294 µg, Y: 278 µg, Z: 727 µg	-	X: 8.7193μg, Y: 9.1956 μg, Z: 30.093 μg
Range	±14 g	±1 g	XY: ±35 g Z: ±20 g	-	±10 g	±1 g	±10 g
Cross-axis coupling errors	-	-	<0.5%	-	<1.5%	<2%	<0.5%
Die	8.7 × 8.7 × 0.5 mm^2^	6 × 6 × 0.6 mm^2^	-		-	6.0 × 5.5 × 1.2 mm^2^	10 × 10 mm^2^
Package	15 × 15 × 5 mm^3^	-	4 × 4 mm^2^		-	-	18 × 18 × 3 mm^3^

## Data Availability

Not applicable.
